# Reaction to Yadav S et al: Invalid presentation of bilateral synchronous spermatocytic seminoma

**DOI:** 10.11604/pamj.2014.19.34.4980

**Published:** 2014-09-15

**Authors:** Kanika Sharma, Piyush Kalakoti, Kush Ohri, Suman Sahu, Sreenivas Veeranki

**Affiliations:** 1 Rural Medical College, Pravara Institute of Medical Sciences, Loni, MH 413736, India; 2 Pushpanjali Crosslay Hospital, Ghaziabad, UP 201012, India; 3 University of Texas Medical Branch, Galveston, TX, United States of America

**Keywords:** Spermatocytic seminoma, synchronous, sequential, testicular tumour

## Reaction

We read with great interest the paper by Yadav and Gupta on bilateral synchronous spermatocytic seminoma (SS) in the recent issue of the *Pan African Medical Journal* [1]. While we commend the authors for their efforts in reporting a rare variant of SS in a 42-year old Indian male, we are of great concern that the authors diagnose it as a bilateral synchronous SS despite the radiographic imaging and intra/post-operative histopathological findings being inadequate and inconsistent with the literature [2, 3]. As Kalakoti and colleagues illustrated in earlier report [2], only 10 cases of SS with bilateral presentation were reported in the medical literature as of mid-February 2014; of them, synchronous presentation of bilateral SS has been ascertained with authority in only five patients using radiographic and/or intra/post-operative histopathological findings [2]. The first case of bilateral synchronous presentation of SS from the Asian subcontinent was reported by Maruta and colleagues [4] in 2011 in a 56-year old Japanese male, almost seven decades since Masson first reported it in 1946 [5]. From the Indian mainland, Koppad and colleagues [2] documented the first such case in a 50-year old male in the year 2012.

Although the possibility of underreporting cannot be ruled out, it is evident that bilateral SS is rare, and the synchronous presentation even rarer. Though not appropriately stated, Yadav and Gupta [1] supposedly claim to have reported the second case of bilateral synchronous SS from India, however it is critical to provide adequate information before such claim be made. The sonographic images presented by the authors illustrate the right testis distinctly, but not bilateral imaging of both testes conjointly. This questions the presentation of a “synchronous” bilateral SS especially with patient's history and sonographic findings possibly inclining towards a “sequential” presentation of bilateral SS or simply a unilateral SS. In addition, the histopathological findings reported by the authors might not be adequate in reporting this case as bilateral synchronous SS since the distinct histopathological characteristics of cellular variation and “anaplastic” intermediate-sized multinucleated cells in both testes are not seen, unlike earlier studies where such findings are clearly evident [3]. Although the diagnositic criteria of bilateral synchronous SS are yet to be established, based on the case details presented by the authors, we opine this case might possibly be a unilateral SS or a bilateral SS with sequential presentation.
